# Exclusive inhibition of PI3K/Akt/mTOR signaling is not sufficient to prevent PDGF-mediated effects on glycolysis and proliferation in colorectal cancer

**DOI:** 10.18632/oncotarget.11899

**Published:** 2016-09-08

**Authors:** Romana Moench, Tanja Grimmig, Vinicius Kannen, Sudipta Tripathi, Marc Faber, Eva-Maria Moll, Anil Chandraker, Reinhard Lissner, Christoph-Thomas Germer, Ana Maria Waaga-Gasser, Martin Gasser

**Affiliations:** ^1^ Department of Surgery I, Molecular Oncology and Immunology, University of Wuerzburg, Wuerzburg, Germany; ^2^ Ribeirao Preto Pharmaceutical Sciences School, Department of Toxicology, Bromatology, and Clinical Analysis, University of Sao Paulo, Sao Paulo, Brazil; ^3^ Brigham and Women's Hospital, Transplant Research Center, Harvard Medical School, Boston, MA, USA; ^4^ Department of Surgery I, University of Wuerzburg, Wuerzburg, Germany

**Keywords:** PDGF, colorectal cancer, PI3K/Akt/mTOR, MAPK pathway, glucose metabolism

## Abstract

Platelet-derived growth factor (PDGF) and signaling via its receptors plays a crucial role in tumor cell proliferation and thus may represent an attractive target besides VEGF/EGFR-based antibody therapies. In this study we analyzed the influence of PDGF in colorectal cancer. PDGF was expressed intensively in early and even more intensively in late stage primary CRCs. Like VEGF, PDGF enhanced human colon cancer proliferation, and increased oxidative glycolytic activity, and activated HIF1α and c-Myc *in vitro*. PDGF activated the PI3K/Akt/mTOR pathway while leaving MAPK signaling untouched. Further dissection showed that inhibition of Akt strongly impeded cancer cell growth while inhibition of PI3K did not. MAPK analysis suggested an inhibitory crosstalk between both pathways, thus explaining the different effects of the Akt and PI3K inhibitors on cancer cell proliferation. PDGF stimulates colon cancer cell proliferation, and prevents inhibitor induced apoptosis, resulting in tumor growth. Therefore inhibition of PDGF signaling seems to be a promising target in colorectal cancer therapy. However, due to the multifaceted nature of the intracellular PDGF signaling, careful intervention strategies are needed when looking into specific signaling pathways like PI3K/Akt/mTOR and MAPK.

## INTRODUCTION

Intensive research has been focused on new treatment strategies in colorectal cancer. Primarily VEGF (Vascular Endothelial Growth Factor), EGF (Epidermal Growth Factor) and their receptors [1–4] have been screened as potential therapeutic targets. As a result, inhibitory monoclonal antibodies as well as tyrosine kinase inhibitors have shown promising outcome in therapy of colorectal cancer (CRC) patients [5]. For example, the monoclonal antibody Bevacizumab intercepts VEGF before binding to his VEGF receptor and thus inhibits activation of intracellular signaling, e.g. the PI3K/Akt/mTOR pathway, and angiogenesis [1, 2]. Besides VEGF, recent studies indicated that also the Platelet Derived Growth Factor (PDGF) influences the tumor cell proliferation and metabolism [6].

Possible downstream signaling pathways targeted by PDGF are the PI3K/Akt/mTOR pathway [7] and the MAPK pathway [8] - maintaining diverse crosstalk points with each other [9, 10]. A PDGF-induced activation of the PI3K/Akt/mTOR pathway influences not only growth, proliferation, and survival, but also shifts in energy metabolism, biosynthesis of macromolecules, and cell cycle progression [11–13].

The downstream target of PI3K/Akt/mTOR signaling, hypoxia-inducible-factor 1α (HIF1α), can influence glucose metabolism not only under hypoxic but also under normoxic conditions [14]. HIF1α triggers both, VEGF secretion, [15] and the expression of glycolytic markers which exert influence on the glycolytic rate in tumor cells. This leads to the synthesis of macromolecules and increased tumor cell proliferation (Warburg effect) [16, 17].

As demonstrated in various studies, PDGF and its receptors were expressed on tumor cells and in surrounding tissue (stroma and pericytes) in colon cancer [18–21]. This suggests not only paracrine but also autocrine PDGF stimulation of cancer cells, which can boost proliferation, supporting the direct effects of PDGF on tumor cells [22].

To understand the impact of PDGF in CRC, the purpose of this study was to elucidate the influence of PDGF on intracellular signaling, proliferation, and cell metabolism in colorectal cancer, and in particular on the PI3K/Akt/mTOR.

## RESULTS

### PDGF and VEGF expression in primary human colon cancer and in colon cancer cell lines

**Figure 1:**
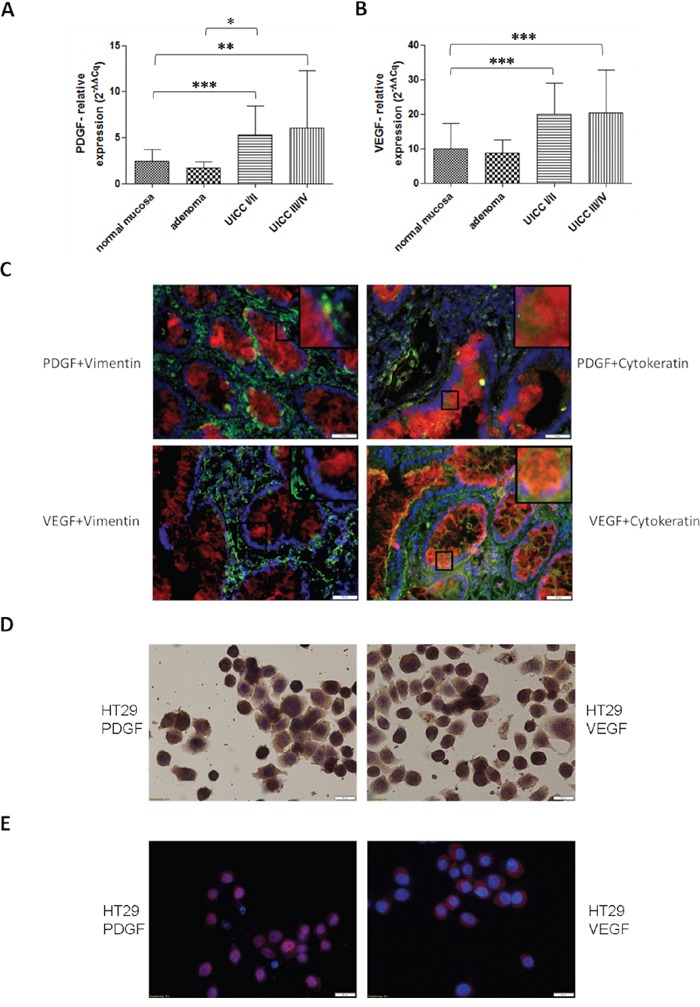
PDGF and VEGF expression in human colon cancer probes and in HT29 cell line Significantly increased gene expression of PDGF **A.** and VEGF **B.** in human colon cancer probes in UICCI/II and III/IV. Normalization was performed with normal 2^−ΔΔCq^. *p<0.05, **p<0.01, ***p<0.001. **C.** Immunofluorescence double staining of colon cancer tissue UICC III/IV exhibited a positive expression of PDGF (Cy3, red) and VEGF (Cy3, red) in epithelial (Cytokeratin, Alexa 488, green) cancer tissue but no or occasionally low expression in stromal regions (Vimentin, Alexa 488, green) with PDGF or VEGF. Magnification x20. **D.** Cytospin of HT29 cells with positive PDGF or VEGF staining. **E.** Immunofluorescence staining showed a positive expression of PDGF (Cy3, red) and VEGF (Cy3, red), nuclear counterstaining with DAPI blue.

To analyze the fundamental role of PDGF in CRC, we first investigated PDGF and VEGF gene expression. Both PDGF and VEGF were significantly overexpressed in CRC samples compared to normal mucosa (p<0.001) (Figure 1A and 1B), with PDGF showing a tendency of increased expression in advanced tumors compared to earlier stage cancers (UICC III/IV vs. UICC I/II). The immunofluorescence double staining in colon cancer cells (UICCIII/IV) with PDGF in combination with Cytokeratin illustrated a clear PDGF expression in epithelial colon cancer cells while PDGF expression in the stromal region (Vimentin staining) was considerably lower or absent. VEGF exhibited the same positive expression in the colon cancer tissue, whereas the expression intensity of PDGF was comparable with the VEGF expression intensity (Figure 1C). DAB- and immunofluorescent analysis of HT29 colon cancer cells also demonstrated tumor cell related expression of VEGF and PDGF (Figure 1D and 1E).

### Influence of PDGF stimulation and Akt-pathway inhibition on proliferation in HT29 cells

**Figure 2:**
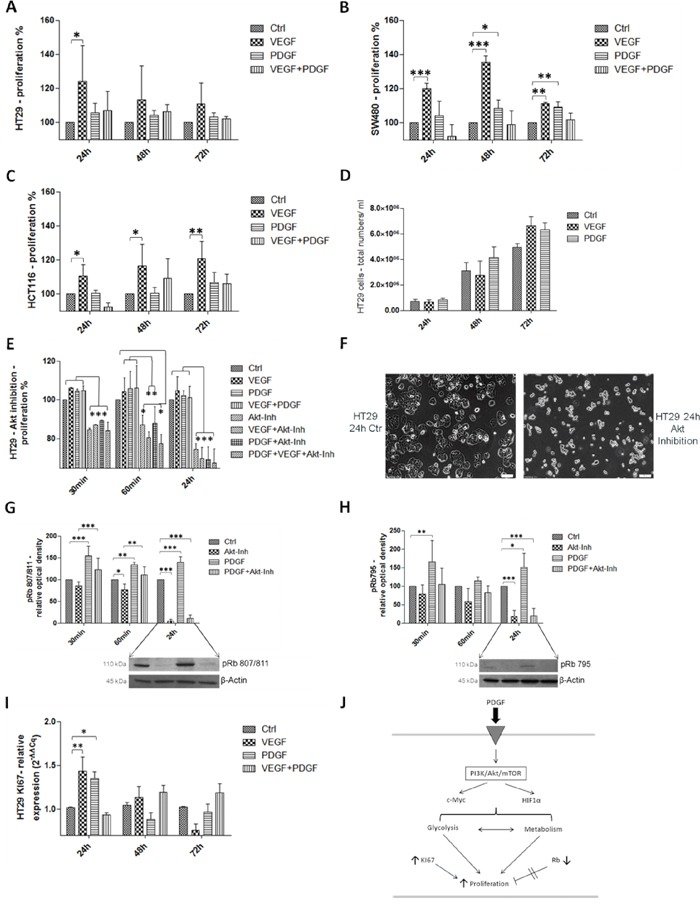
Influence of PDGF stimulation and Akt-pathway inhibition on proliferation in HT29, SW480, and HCT116 J PDGF and VEGF stimulation enhanced cell proliferation in the MTS assay in HT 29 **A.**, SW480 **B.**, and HCT116 cells **C.** **D.** Increased cell number after PDGF and VEGF stimulation (PDGF and VEGF stimulation 100 ng/ml, new stimulation every 24h). **E.** Akt inhibition (10 μM) induced a decreased proliferative effect in the MTS assay in HT29 cells. **F.** show the antiproliferative effect of the Akt inhibition compared with untreated control cells. **G** and **H.** Lower panels showed representative western blots and upper panels showed quantification of three independent western blot experiments of pRb (Ser 807/811) (G), and pRb (Ser 795) (H), normalized to β-Actin loading control. The tumor suppressor Rb was inactivated during PDGF stimulation and activated during Akt inhibition. **I.** The proliferation marker KI67 was increased on gene level during PDGF and VEGF stimulation. *p<0.05, **p<0.01, ***p<0.001.

MTS proliferation analysis (Figure 2J) of HT29 cells confirmed an increased proliferative response after both PDGF- and VEGF-stimulation (p<0.05 after 24h). Combined stimulation with PDGF and VEGF showed only partial synergistic effects compared to control (Figure 2A). Additional MTS assays performed with SW480 (Figure 2B) and HCT116 (Figure 2C) colon cancer cells showed similar influences of PDGF (SW480: p<0.05 (48h); p<0.01(72h)), and VEGF (SW480: p<0.001 (24h and 48h); p<0.01 (72h); HCT116: p<0.05 (24h and 48h); p<0.01 (72h)) on tumor cell proliferation. After 72 hours of stimulation with PDGF and/or VEGF, HT29 cells showed a higher cell number in the cell count compared with unstimulated controls (Figure 2D). Gene expression of the proliferation marker KI67 was also increased in these cell lines (HT29: Figure 2I, HCT116: supplementary Figure S5A, and SW480: supplementary Figure S5B), which confirmed an accelerated cell cycle and an increased proliferative effect provoked by PDGF and VEGF, primarily after 24 hours.

To check the proliferative influence of PDGF on the PI3K/Akt/mTOR pathway, inhibition of the central Akt protein with InSolution™ Akt Inhibitor IV was conducted. Inhibition of Akt significantly reduced cell proliferation (Figure 2E), but 30 minutes after Akt inhibition, treatment with PDGF and VEGF attenuated the anti-proliferative effect caused by the Akt inhibitor. After 24 hours, neither PDGF nor VEGF suppressed the inhibitory effect on cell proliferation (Figure 2E). After 24 hours of incubation with the Akt inhibitor, the morphological appearance of HT29 cells was adversely affected compared with untreated control cells (Figure 2F).

### Effects of PDGF and PI3K/Akt-pathway inhibition on apoptosis

**Figure 3:**
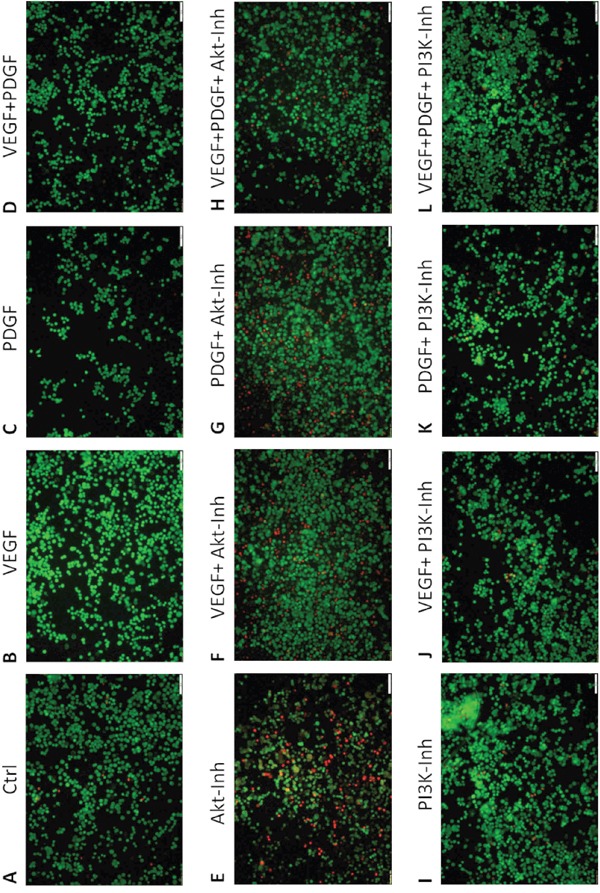
Effect of PDGF stimulation and/or Akt- and PI3K inhibition on apoptosis - Live/Dead Cell staining assay Single treatment with PDGF **C.** VEGF **B.** or PDGF + VEGF **D.** showed decreased amounts of dead cells (red) compared to Akt inhibitor treatment **E.** and control **A.** PDGF and VEGF stimulation **F-H.** decreased apoptosis (living cells, green). PI3K inhibition **I.** did not induce apoptosis, therefore PDGF and VEGF stimulation did not increase cell survival **J-L.** Magnification x10.

Next to tumor cell proliferation, the influence of PDGF and/or PI3K/Akt-pathway inhibition on apoptosis in HT29 colorectal cancer cells was investigated. In the live/dead cell staining assay, PDGF (Figure 3C), VEGF (Figure 3B), and both PDGF and VEGF (Figure 3D) slightly increased the amount of living cells (green staining) compared to untreated HT29 cells (Figure 3A). Furthermore, stimulation (Figure 3F-3H) decreased the amount of dead cells in presence of the Akt inhibitor compared to exclusive Akt inhibition (Figure 3E). The Akt inhibition showed an increased amount of dead cells (read staining) (Figure 3E) compared to control cells (Figure 3A). During PI3K inhibition the proportion of dead cells remained unaltered (Figure 3I). Thus treatment of PDGF and VEGF (Figure 3J-3L) in presence of the PI3K inhibitor (Figure 3I) did not result in an increased survival of cells. The additionally performed ApopTag^®^ Plus apoptosis assay showed comparable results (Supplementary Figure S1).

**Figure 4:**
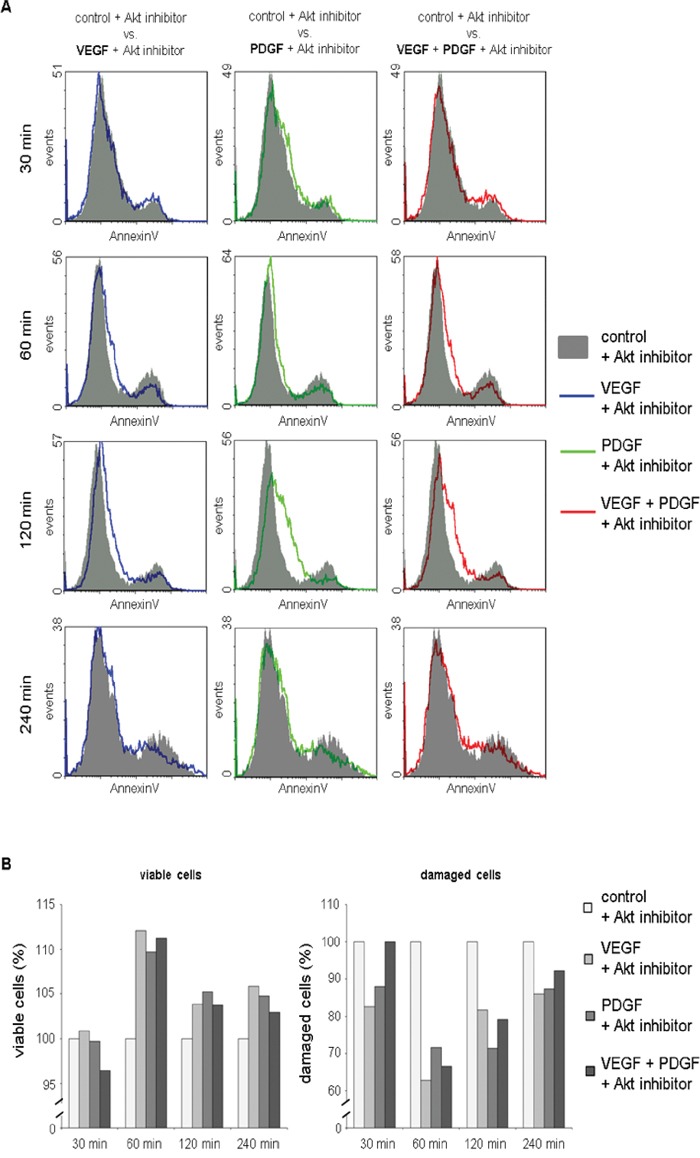
Reduced cytotoxicity of Akt inhibition after additional treatment with PDGF and/or VEGF in AnnexinV apoptosis assay **A.** Overlay histograms of AnnexinV fluorescence in cells treated with Akt inhibitor + VEGF, Akt inhibitor + PDGF, and Akt inhibitor + VEGF + PDGF compared to sole Akt inhibition demonstrated a reduction of dead cells (right peak) in samples supplemented with VEGF and/or PDGF 60 to 240 minutes past treatment. **B.** Proportions of viable cells were found elevated in samples additionally incubated with VEGF and/or PDGF compared to sole Akt inhibition 60 to 240 minutes past treatment. Correspondingly, reduced proportions of damaged cells (apoptotic, necrotic, and dead cells) were detected in samples additionally treated with VEGF and/or PDGF. VEGF + Akt inhibitor blue line, PDGF + Akt inhibitor green line, VEGF + PDGF + Akt inhibitor red line.

In flowcytometric AnnexinV apoptosis analysis, the overlay histograms of HT29 cells demonstrated a reduction of dead cells (right peak) in samples additionally supplemented with VEGF and/or PDGF 60 to 240 minutes past treatment compared to exclusive treatment with the Akt inhibitor (Figure 4A). Additionally, PDGF exhibited a decreased proportion of dead cells compared to VEGF after 120 minutes (Figure 4A). The transition between the peak of the living cells (left peak) and the dead cells (right peak), which represents the proportion of apoptotic cells, showed that PDGF and VEGF increased the proportion of apoptotic cells and reduced the amount of dead cells.

Moreover, proportions of viable cells were found elevated in samples additionally incubated with VEGF and/or PDGF compared to sole Akt inhibition 60 to 240 minutes past treatment. Correspondingly, reduced proportions of damaged cells (apoptotic and necrotic) were detected in samples additionally treated with VEGF and/or PDGF (Figure 4B).

### Influence of PDGF and the Akt-pathway inhibition on tumor suppressor Rb

For a more detailed study of the influence of PDGF on cell cycle and proliferation, the effect of PDGF on the tumor suppressor *Retinoblastoma* (Rb) was investigated (Figure 2J). Rb controls transition of the G1-phase to the S-phase. It encloses the transcription factor E2F, but phosphorylation of Rb causes E2F release and leads to cell cycle progression [23, 24], because only free E2F is able to lead the cell cycle from the G1-phase into the S2-phase.

Stimulation with PDGF provoked increased phosphorylation and thus inactivation of Rb (p<0.01 (60min) and p<0.001 (30 min and 24h) for pRb807/811, and p<0.05 (72h), and p<0.01 (30min) for pRb795). Inhibition of Akt showed a pronounced decline in the phosphorylation status of Rb (p<0.05 (60 min) and p<0.001 (24h) for pRb807/811 and p<0.001 (24h) for pRb795) and therefore more Rb activity, which resulted in more effective cell cycle control. PDGF decreased the effect of the Akt inhibitor (p<0.001 (30min) and p<0.01 (60 min) for pRb807/811), and increased phosphorylation and hence inactivation of Rb (Figure 2G and 2H).

### Effects of PDGF stimulation and Akt inhibition on the PI3K/Akt/mTOR pathway and MAPK pathway in colon cancer

**Figure 5:**
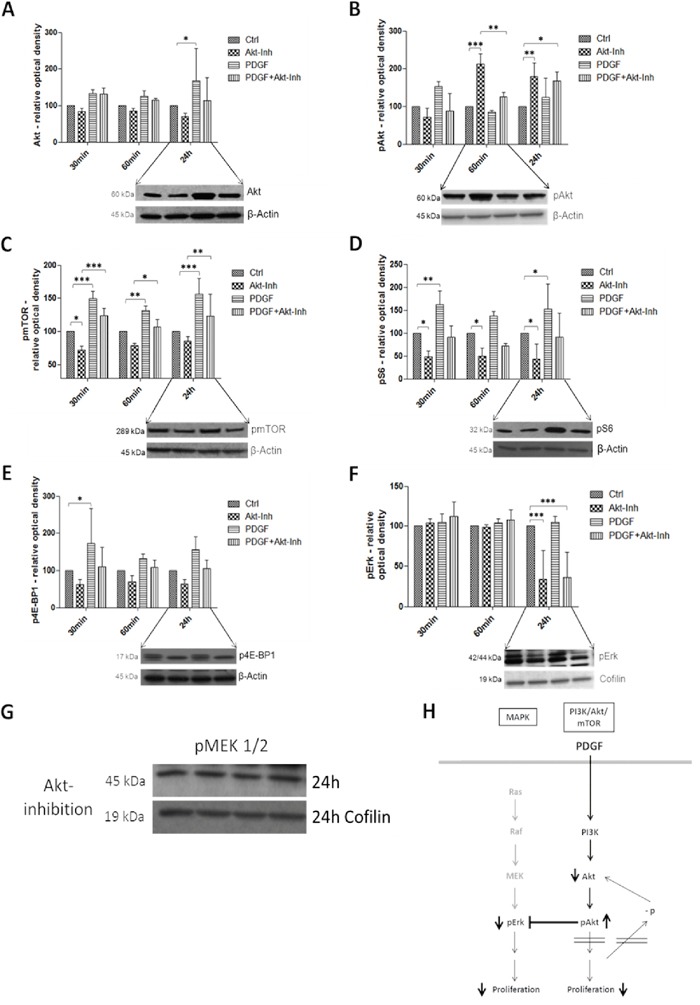
Western Blot analysis showed the effects of PDGF stimulation and/or Akt inhibition on the PI3K/Akt/mTOR and MAPK pathway in HT29 cells H Lower panels show representative western blots and upper panels show quantification of three independent western blot experiments of Akt **A.**, pAkt **B.**, pmTOR **C.**, pS6 **D.**, p4E-BP1 **E.**, and pErk **F.**, normalized to Actin or Cofilin loading control. Cells were treated with Akt Inhibitor IV (10 μM) or PDGF (100 ng/ml), and with both Akt inhibitor and PDGF. Results are presented as ±SD. *p<0.05, **p<0.01, ***p<0.001. Representative western blots of pMEK 1/2 **G.** during Akt inhibition (loading control Cofilin).

To investigate PDGF induced influence on the PI3K/Akt/mTOR pathway, we first used a specific Akt inhibitor (InSolution™ Akt Inhibitor IV) (Figure 5H). Due to the inhibition of Akt, Akt protein expression was inactivated, but activated by PDGF (p<0.05) (Figure 5A). Synchronous inhibition and stimulation of HT29, HCT116 (Supplementary Figure S2), and SW480 (Supplementary Figure S3) cells increased protein expression of Akt, compared to control and inhibitor, but decreased activity compared with exclusive stimulation with PDGF. Expression analysis of phosphorylated and thereby activated Akt (pAkt) showed the same results after a 30-minute treatment. pAkt was deactivated during Akt inhibition, and upregulation was caused by stimulation with PDGF. However, after 60 minutes a reverse effect was observed. pAkt activity was significantly increased (p<0.001) (Figure 5B), and Akt was decreased by initiating Akt inhibition. The now-onset inhibition of Akt directly below pAkt in the downstream signaling cascade provoked reduced Akt protein and elevated pAkt protein expression (Figure 5A and 5B). Stimulation with PDGF resulted in a decreased pAkt and increased Akt (p<0.05) protein expression, by means of active PI3K/Akt/mTOR signaling. PDGF mitigated the Akt inhibition and increased the PI3K/Akt/mTOR pathway activity (Figure 5B).

mTor (mammalian of rapamycin), S6 (S6 ribosomal protein), and 4E-BP1 (eukaryotic translation initiation factor 4E binding protein 1, p4E-BP1) are downstream targets of Akt. The translation repressor 4E-BP1 binds to eIF-4E (eukaryotic translation initiation factor 4E) and inhibits translation, protein synthesis, and proliferation. Phosphorylation, caused by mTOR, inactivated 4E-BP1 [25–27]. mTOR and pS6 were inhibited by Akt inhibition (p<0.05), but activated after stimulation with PDGF (p<0.01 and p<0.001 respectively) (Figure 5C and 5D). 4E-BP1 was dephosphorylated and thus translation was inactivated by Akt inhibition, but stimulation with PDGF increased the inactive, phosphorylated version of 4E-BP1 (p<0.05) (Figure 5E). Combined inhibition and activation showed a higher activity of pS6, p4E-BP1, and pmTOR (p<0.01 after 24 hours) than sole Akt inhibition in the colon cancer cell lines HT29, HCT116 and SW480 (exception: SW480 cells showed a reverse 4E-BP1expression pattern, Supplementary Figure S3E)

pErk, downstream target of the MAPK pathway [28], was significantly inactivated by Akt inhibition after 24 hours (p<0.001). However, stimulation with PDGF did not activate pErk (Figure 5F). Interestingly, pMEK1/2, also part of the MAPK signaling but upstream of Erk, was not influenced by both PDGF and the Akt inhibitor (Figure 5G).

### Effects of PDGF stimulation and PI3K inhibition on the PI3K/Akt/mTOR and MAPK pathway in colon cancer

**Figure 6:**
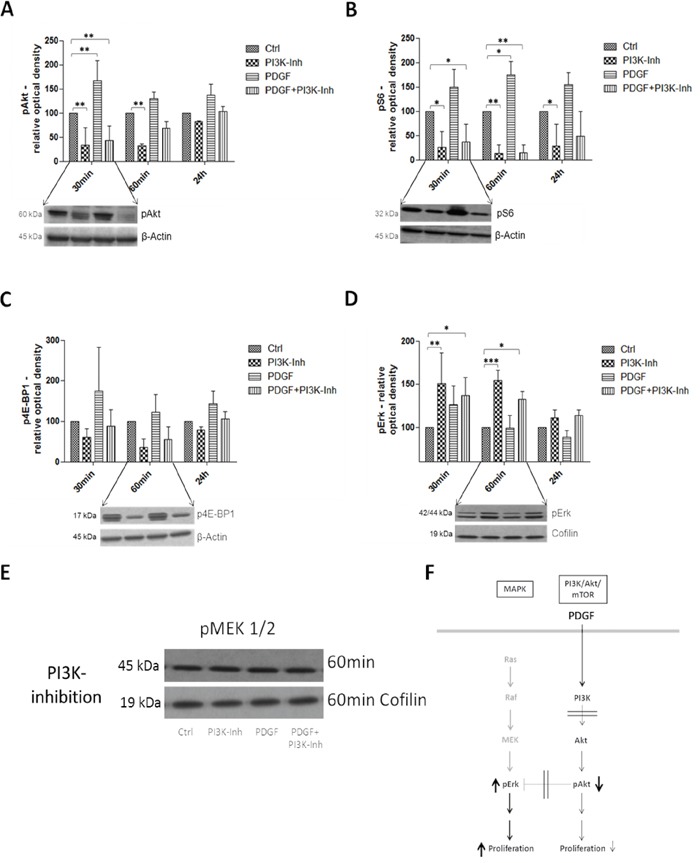
Western Blot analysis representing the effects of PDGF stimulation and/or PI3K inhibition on the PI3K/Akt/mTOR and MAPK pathway in HT29 cells F Lower panels show representative western blots and upper panels show quantification of three independent western blot experiments of pAkt **A.**, pS6 **B.**, p4E-BP1 **C.**, and pErk **D.**, normalized to Actin or Cofilin loading control. Cells were treated with PI3K Inhibitor (80 nM) or PDGF (100 ng/ml), and with both PI3K inhibitor and PDGF. Results are presented as ±SD. *p<0.05, **p<0.01, ***p<0.001. **E.** Representative western blot of pMEK 1/2 during PI3K inhibition (loading control Cofilin).

The second analyzed inhibition target of the PI3K/Akt/mTOR pathway was PI3K in HT29, HCT116 (Supplementary Figure S4A-S4D), and SW480 (Supplementary Figure S4E-S4H) colon cancer cells. Unlike the Akt inhibition, and as expected, pAkt was significantly (p<0.01) inhibited after 30 minutes and further on after 60 minutes (p<0.01) by the PI3K inhibitor (Figure 6A). pS6 and p4E-BP1 activity was likewise suppressed, particularly after 30 minutes and 60 minutes respectively (Figure 6B and 6C). Both targets were activated by stimulation with PDGF; parallel inhibition and stimulation showed higher expression than treatment with the PI3K inhibitor alone. Interestingly, pErk was significantly activated (p<0.01 after 30minutes and p<0.001 after 60minutes) by the PI3K inhibition (Figure 6D). pMEK, again, remained unmodified by PDGF and PI3K inhibition (Figure 6E).

### Influence of PDGF on metabolism/glycolysis in colon cancer tissues

**Figure 7:**
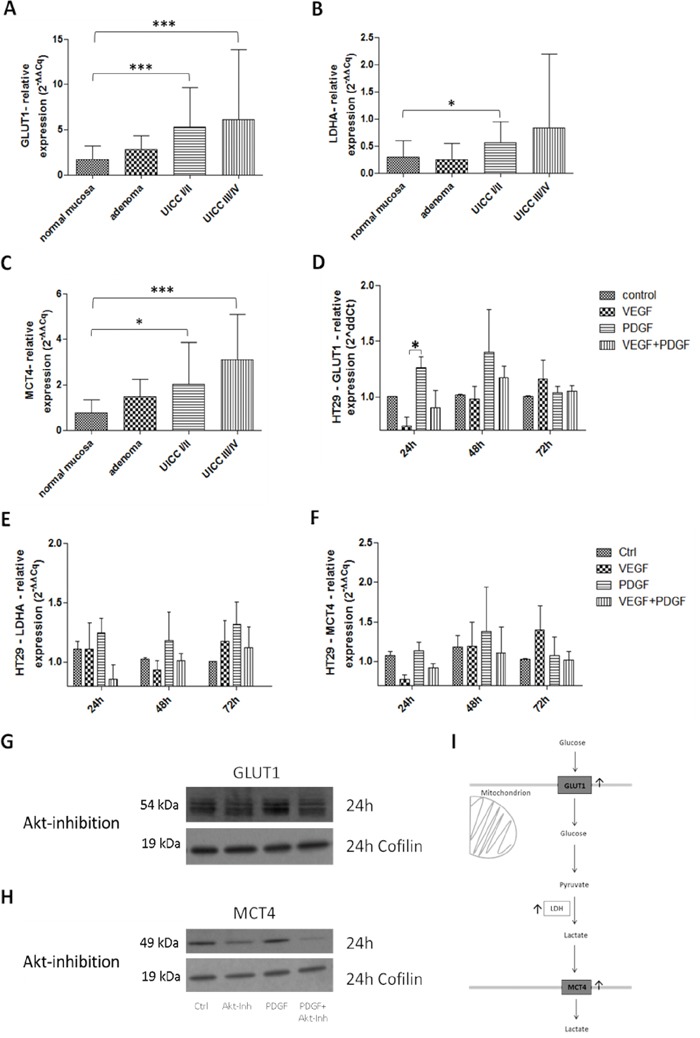
Influence of PDGF on glycolysis in human colon cancer probes and in HT29 cell line I Gene expression of the glycolysis markers GLUT1 **A.**, LDHA **B.**, and MCT4 **C.** was activated on gene level at stage UICC III/IV in the human colon cancer probes. Results were presented as ±SD. *p<0.05, ***p<0.001. **D-F.** HT29 cells were treated with PDGF or VEGF or both PDGF and VEGF (100 ng/ml respectively) for 24 hours, 48 hours, and 72 hours. The glycolysis markers GLUT1 (D), LDHA (E), and MCT4 (F) were activated on gene level mainly during PDGF stimulation but also during VEGF stimulation. Representative western blots of GLUT1 **G.**, and MCT4 **H.** during Akt inhibition (loading control Cofilin), n=3.

In primary colon cancer, a constitutively increased mRNA expression of the glycolysis markers GLUT1, LDHA, and MCT4 (Figure 7I) was observed. GLUT1 was significantly augmented in all tumor stages (p<0.001) (Figure 7A); LDH also showed a higher expression in all stages (Figure 7B). MCT4 exhibited a significantly higher expression in particular at advanced stages (p<0.001) (UICC III/IV) compared to controls, and patients with colon adenomas (Figure 7C).

After 24 hours of initial stimulation with PDGF, GLUT1 showed a significantly increased expression (p<0.05) in HT29 cells (Figure 7D). Increased LDHA and MCT4 gene expression followed after 48 hours and 72 hours upon stimulation with PDGF (Figure 7E and 7F). Stimulation with VEGF provoked an attenuated expression of GLUT1, LDHA and MCT4, compared to controls, but only after 72 hours of stimulation. HCT116 and SW480 cells exhibited the same expression patttern (Supplementary Figure S5C-S5H). GLUT1 and MCT4 activity were reduced during Akt inhibition on protein level in HT29 cells (Figure 7G and 7H).

### Influence of PDGF on oxygen consumption, glucose and lactate transport, and mitochondrial comlexes in HT29 colon cancer cells

**Figure 8:**
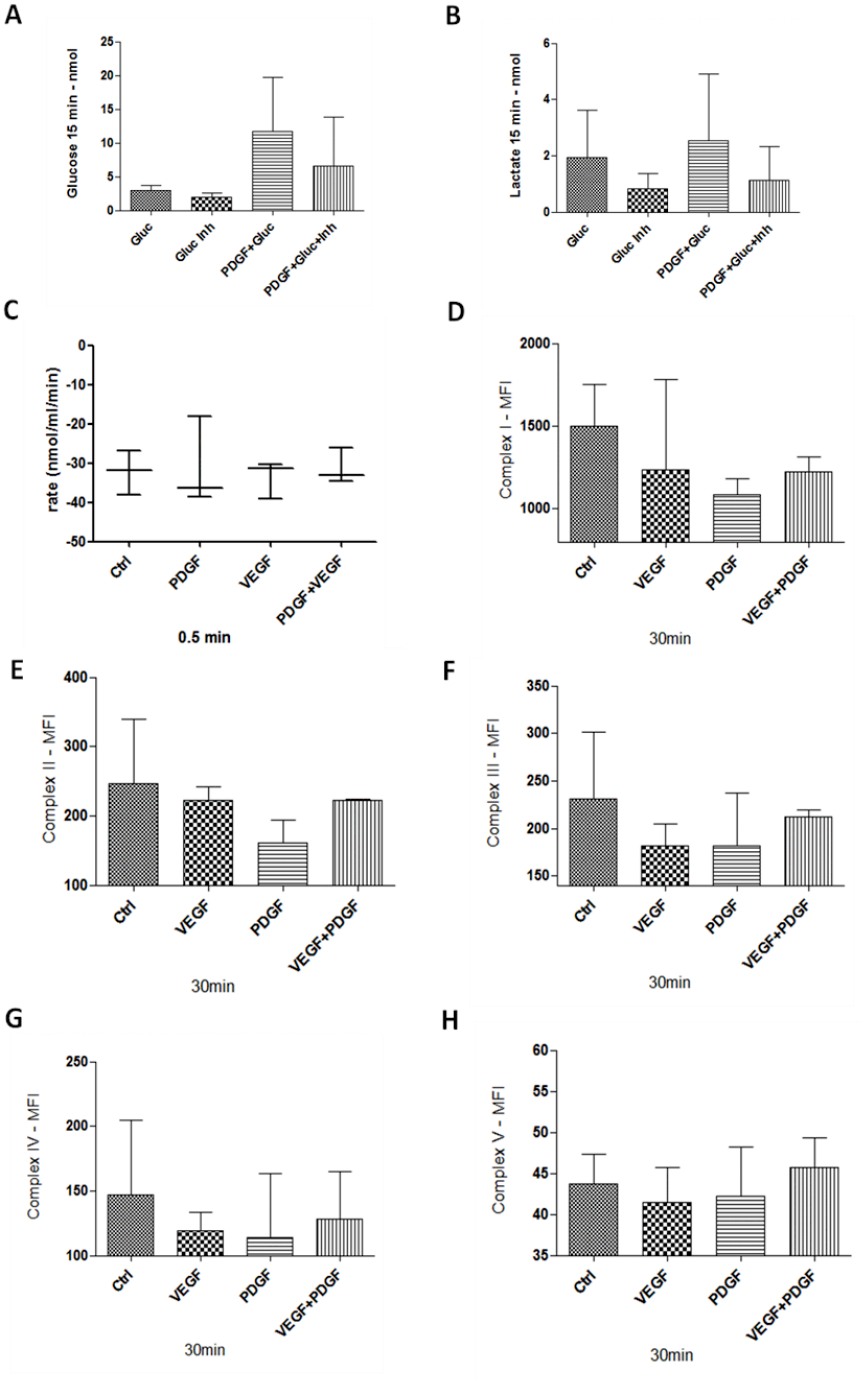
Glucose and Lactate measurement, Oxygen consumption, and mitochondrial complexes activity in HT29 cells **A** and **B.** 1 × 10^6^ cells were stimulated with PDGF (100 ng/ml) for 24 hours. Unstimulated cells and cells treated with PDGF were washed with glucose-free buffer (110mM NaCl, 5mM KCl, 1mM MgCl_2_, 4mM Na_2_PO_4_, 50mM Na-HEPES, pH 7.4), and treated with Glucose (20mM), GLUT1 inhibitor WZB117 (10 μM) or both. Glucose uptake (A), and lactate release (B) was measured with an abcam Glucose and Lactate Assay. HT29 cells were treated with PDGF or VEGF or both PDGF and VEGF (100 ng/ml) for 24 hours, n=3. Oxygen consumption **C.** showed no changes during PDGF and/or VEGF stimulation. For mitochondrial activity measurement **D-H.**, cells were treated with PDGF, VEGF or both for 30 minutes, and measured with a MILLIPLEX^®^ human oxidative phosphorylation (OXPHOS) magnetic bead panel, n=2. PDGF and VEGF decreased the activity of the mitochondrial complexes.

The use of glucose transporter 1 (GLUT1) inhibitor IV, WZB117, caused a decreased glucose uptake into HT29 colon cancer cells, whereas a higher glucose influx was detected during stimulation with PDGF (Figure 8A). Interestingly, during PDGF influence the glucose uptake of colon cancer cells was increased, even in presence of the GLUT1 inhibitor. Release of lactate, as a product of glycolysis, was reduced during GLUT1 inhibiton, and enhanced during stimulation with PDGF (Figure 8B). Stimulation with PDGF caused higher glucose uptake into tumor cells and increased lactate release after 15 minutes of treatment (Figure 8A and 8B).

While PDGF stimulated glucose and lactate metabolism, oxygen consumption during stimulation with PDGF and VEGF remained unchanged (Figure 8C).

Measurement of the mitochondrial complexes I-V revealed a loss of activity during stimulation with PDGF and VEGF, compared to unstimulated tumor cells, at which PDGF showed a stronger inhibitory effect compared to VEGF (Figure 8D-8H). These data indicate a decreased mitochondrial activity and an increased glycolysis during PDGF stimulation.

### Investigation of HIF1α and c-Myc in colon cancer

**Figure 9:**
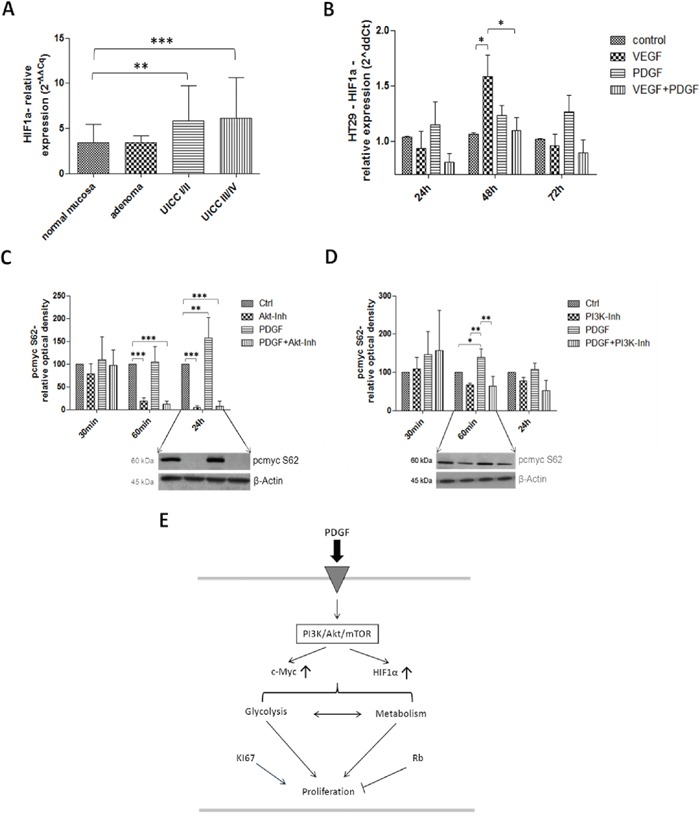
Investigation of HIF1α in human colon cancer probes (A), and in HT29 cells (B) and c-Myc (C and D) in HT29 cells (E) Gene expression of HIF1α was increased on gene level at stage UICC III/IV in the human colon cancer probes **A.** and during PDGF- and VEGF-stimulation (100 ng/ml) in HT29 cells **B.** **C** and **D.** Lower panels show representative western blots and upper panels show quantification of three independent western blot experiments of pc-Myc (S62), normalized to β-Actin loading control. Pc-Myc activity was increased by PDGF, and decreased by Akt inhibitor. Cells were treated with PDGF (100 ng/ml), Akt inhibitor IV (10 μM) (C) or PI3K Inhibitor (80 nM) (D). Results are presented as ±SD, *p<0.05, **p<0.01, ***p<0.001.

HIF1α and c-Myc take central positions in glycolysis regulation and thus in proliferation (Figure 9E). In primary colon tumors of patients, especially at advanced stages (UICC III/IV), a significantly higher gene expression of HIF1α (p<0.001) was observed (Figure 9A). Stimulation with PDGF also resulted in increased HIF1α expression in HT29 colon cancer cells, which was further intensified after stimulation with VEGF (p<0.05) after 48 hours (Figure 9B).

Activated c-Myc was significantly suppressed during Akt inhibition (p<0.001) in HT29 cancer cells (after 60 minutes and 24 hours of incubation), but PDGF significantly increased the c-Myc activity after 24 hours of stimulation (p<0.01) (Figure 9C). During PI3K inhibition, similar but attenuated effects were visible during stimulation with PDGF (after 60 minutes: p<0.05 to control; p<0.01 to PI3K inhibition, and to PDGF+PI3K inhibition), and during Akt inhibition (Figure 9D).

## DISCUSSION

### PI3K/Akt/mTOR and MAPK pathway

Recent studies provide growing evidence of the important role of PDGF in colorectal cancer [29]. However, the influence of PDGF in colorectal cancer appears to be multifactorial and not completely understood in all aspects. We analyzed the PDGF expression in human colon cancer and dissected its role upon stimulation in colon cancer cells. We also compared the effects of PDGF with those from VEGF for most of the experiments.

**Figure 10:**
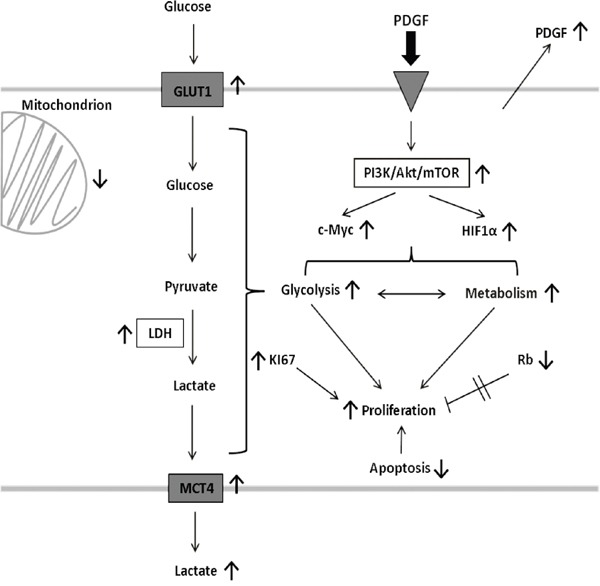
Effects of PDGF on tumor cell signaling, and metabolism PDGF decreases apoptosis, and increases glycolysis and proliferation in an Akt-dependent manner in CRC. It also activates HIF1α, and C-Myc, and suppresses Rb to accelerate metabolism and the proliferative potential of PDGF. This stimulating mechanisms highlight the importance and the potential of the PDGF-PI3K/Akt/mTOR pathway-axis as a possible target in colorectal cancer. PDGF, platelet-derived growth factor; PI3K, phosphoinositide 3-kinase; mTOR, target of Rapamycin; HIF1α, hypoxia-inducible-factor 1α; Rb, Retinoblastoma; GLUT1, glucose transporter 1; LDHA, lactate dehydrogenase A; MCT4, monocarboxylate transporter 4.

In human colon cancer, PDGF and VEGF were highly expressed in all stages (UICC I-IV), which underlined an involvement of both PDGF and VEGF in tumor progression. HT29 cancer cells also secreted VEGF, and PDGF, which suggested an autocrine stimulation of the cancer cells (Figure 10). Based on this, we felt a precise investigation of the role of PDGF on colon cancer cells was appropriate.

Stimulation with both, PDGF and VEGF, showed an increased proliferative response in HT29 colon cancer cells, whereby VEGF exhibited a greater effect on proliferation. However, the combined stimulation with PDGF and VEGF showed no synergistic effects, which suggests a competitive mechanism in the cells [30].

Possible influences of PDGF on proliferation could be an activating effect of PDGF on the PI3K/Akt/mTOR pathway and a supplementary inhibition of the tumor suppressor Retinoblastoma (Rb), and thus activation of the cell cycle. Simultaneously, an activation of Rb was detectable during PI3K/Akt/mTOR pathway inhibition, reinforcing the antiproliferative effect of Rb [31]. Stimulation with PDGF induced an activation of the PI3K/Akt/mTOR pathway, while the MAPK pathway was unaffected by its stimulation, which confirms other results [32]. This showed that PDGF operates through the PI3K/Akt/mTOR pathway and not through the MAPK pathway in CRC.

Interestingly, the two inhibiting substances, Akt inhibitor IV (Akt inhibitor) and PKI-179 (PI3K inhibitor), showed different effects on HT29, HCT116, and SW480 cancer cell proliferation. While the Akt inhibitor demonstrated dramatic antiproliferative effects on these cells, the PI3K inhibitor revealed only marginal effects on cell proliferation.

It is well known that the PI3K/Akt/mTOR and the MAPK pathways are connected via crosstalk bonds [9, 10, 33]. Further investigation of the MAPK pathway demonstrated the specific inhibitory crosstalk [10] bond between PI3K/Akt/mTOR pathway, namely the Akt and MAPK pathway, primarily Erk. Our protein analysis after Akt inhibition showed an activation of pAkt and downregulation of pmTOR, pS6 and p4E-BP1, downstream targets of Akt [34, 35]. This resulted in identification of the specific inhibition position found directly downstream after Akt phosphorylation. The Akt inhibitor caused an increased phosphorylation of Akt after 60 minutes, but the signal was blocked downstream, and caused a visible downregulation of pS6, pmTOR, and p4E-BP1 protein expression after 24 hours. pErk activity was also suppressed, because the inhibitory crosstalk was still active due to the boosted Akt phosphorylation. The three investigated cell lines showed consistency in the results, only 4E-BP1 in the SW480 cells exhibited an opposite protein expression. The earlier Dukes' stage of SW480 (SW480: Dukes' B, HT29: Dukes' C, HCT116: Dukes' D), and thus an altered pattern of mutations, and the different MSI instability [36] could be responsible for this deviation. Despite this high mutational heterogeneity the results did not appear to be cell-specific, but common in colon cancer. This specific Akt inhibitor allows inhibition of both PI3K/Akt/mTOR and MAPK pathway at once, and subsequently inhibition of cancer cell proliferation (Figure 5H).

PI3K inhibition showed an expected downregulation of pAkt and an increased activity of pErk because of the removal of the crosstalk inhibition of Akt. The MAPK pathway seemed to compensate the reduced proliferation of the PI3K/Akt/mTOR pathway during PI3K-inhibition (Figure 6F). Our data support the necessity to inhibit both, the PI3K/Akt/mTOR, and the MAPK pathway. But single inhibition of the PI3K/Akt/mTOR pathway at the right location would be preferable to induce significant tumor cell damage, as inhibition of both pathways may otherwise cause additional clinical side effects. Therefore, future studies should focus on finding a PI3K/Akt/mTOR-specific inhibitor that also inhibits compensatory crosstalk with the MAPK pathway.

### Apoptosis and cell death

To closer investigate the morphological changes and dramatic antiproliferative effect of HT29 cells during Akt inhibition, cell death and apoptosis were examined. During Akt inhibition, HT29 cells exhibited not only cell death, indicating morphological changes. They also exhibited an increased proportion of dead cells, demonstrating the apoptotic influence of the Akt inhibitor. On the other hand, PDGF, VEGF, and both PDGF and VEGF, decreased the proportion of dead cells in presence of the Akt inhibitor compared to exclusive inhibition. These data indicate an apoptosis-preventing effect of PDGF; the PI3K inhibitor marginally increased the proportion of dead cells.

PDGF also appeared to have a cytoprotective effect on HT29 cells during Akt inhibition. In the AnnexinV analysis, stimulation with PDGF and VEGF increased the proportion of both living cells and apoptotic/necrotic cells. PDGF and VEGF, correspondingly, decreased the amount of dead cells despite Akt inhibition; particularly PDGF, but also VEGF delayed HT29 cell death. Combined stimulation with PDGF and VEGF exhibited a similar cytoprotective effect than sole PDGF stimulation, an indication that PDGF possessed a stronger anti-apoptotic effect. Percentage analyses of the living/dead cell ratio also clarified the anti-apoptotic potential of PDGF and VEGF: their cytoprotection increased the proportion of living cells and reduced the proportion of damaged cells (apoptotic, necrotic, and dead cells), in comparison to sole Akt inhibition.

### Glucose metabolism

Stimulation with PDGF can provoke changes in metabolism of colon cancer cells by activation of the PI3K/Akt/mTOR pathway [16]. Therefore the consequences of PDGF on glycolysis were investigated. The glucose transporter 1 (GLUT1) regulates glucose uptake [16, 37, 38], the lactate dehydrogenase A (LDHA) converts pyruvate to lactate [16, 38–40] and the monocarboxylate transporter 4 (MCT4) controlls release of lactate [16, 38, 41] (Figure 10). In many tumor cells the glycolytic shift towards “oxidative glycolysis” was strongly activated, instead of metabolizing glucose by oxidative phosphorylation (Warburg effect) [38, 42, 43] to produce important biosynthetic intermediates for cell proliferation and hence tumor growth [16]. In HT29 colon cancer cells, all three targets (GLUT1, LDHA and MCT4) were activated, and increased glucose uptake and lactate release by stimulation with PDGF was detectable. The data indicated a higher glycolytic rate in presence of oxygen, while oxygen consumption remained constant. The colon cancer cells regulate their energy metabolism more flexibly in the presence of PDGF. Unlike proliferation, where VEGF took the dominant role, PDGF instead seems to play the greater role in the conversion to malignant metabolism. These data suggest the posibility that PDGF and VEGF can act in a synergistic manner for accelerated cancer progression.

Primary human colon cancers demonstrated a significant activation of GLUT1, LDHA, and MCT4 gene expression, which supports the importance of glycolysis in colon cancer, particularly at late stages. Interfering with the PDGF-induced acceleration of glycolysis represents a potential target in tumor metabolism in all progression stages (Figure 10).

### HIF and c-Myc

HIF1α, an important transcription factor, upregulates glycolytic enzymes like GLUT1 [16], LDHA [44, 45], and MCT4 [46]. Importantly, HIF1α expression can be activated by the PI3K/Akt/mTOR pathway [16, 38]. It is stabilized under hypoxia and supports the Warburg effect [16, 47]. In tumor cells it can be activated also under normoxic conditions (“pseudohypoxia”) [14, 48, 49].

The transcription factor and potential oncogene c-Myc influences proliferation, cell cycle, and cellular metabolism. c-Myc is overexpressed by the majority of colon cancers [50–52], and the human HT29 cancer cell line is known as c-Myc-positive. It is also known that c-Myc and HIF1α are able to collaborate with each other with great influence on aerobic glycolysis [45, 51, 53].

Investigation of HIF1α in human colorectal cancers of the patient cohort showed an increased expression in all tumor stages (UICC I-IV). In HT29 cancer cells, stimulation with PDGF and VEGF activated HIF1α; c-Myc was more activated during stimulation with PDGF and the inhibition of Akt showed a decrease in c-Myc activity. Obviously, PDGF increased activity of the HIF1α-c-Myc axis.

Akt inhibition reduced c-Myc activity, because both PI3K/Akt/mTOR and MAPK pathways were inhibited. The PI3K inhibitor, however, showed only moderate inhibitory effects on c-myc; in this case the MAPK pathway was activated due to the reverse inhibitory crosstalk between PI3K/Akt/mTOR and MAPK pathway.

The oncogenic potential of the transcription factors c-Myc and HIF1α has great influence on tumor energy metabolism and triggers tumor progression [54]. In this study, both HIF1α and c-Myc seemed to be related to PDGF and the PI3K/Akt/mTOR pathway (Figure 8). Inactivation of PI3K/Akt/mTOR led to reduced c-Myc, GLUT1, and MCT4 activity. PDGF mediated activation of PI3K/Akt/mTOR, and subsequent activation of HIF1α and c-Myc, resulting in activation of GLUT1, LDHA, and MCT4. This contributes to increased aerobic glycolysis/Warburg effect [14, 15, 17, 50, 53, 55] and proliferation in CRC.

In conclusion, PDGF activates the PI3K/Akt/mTOR pathway, and suppresses apoptosis, even in presence of the Akt inhibitor. PDGF also activates HIF1α, and c-Myc, increases glycolysis and suppresses the tumor suppressor Rb, resulting in modified tumor metabolism and increased proliferation. The anti-apoptotic, pro-metabolic, and pro-proliferating effect of PDGF catalyzes colorectal cancer progression (Figure 10).

More in-depth investigations of specific crosstalk connections between signaling pathways need to be performed. The use of targeted therapy in CRC needs to be highly selective to block possible escape mechanisms amongst the intracellular signal pathways efficiently. A better understanding of pathway crosstalks should be the aim of future studies to negate possible effects of “over inhibition” of pathways, resulting in clinical side effects in patients. Our data highlight the importance of the PDGF-PI3K/Akt/mTOR pathway-axis and its potential as a possible weak point in colorectal cancer.

## MATERIALS AND METHODS

### Tissue samples

Colon cancer tissue samples, and corresponding normal mucosa samples were obtained from 46 patients (5 adenoma, 19 patients UICC I/II, 22 patients UICCIII/IV), undergoing curative surgical resection in our surgical department between 09/2009 and 05/2013. From all patients an informed consent was obtained before tissues were collected. Ethical approval was obtained from the Human Research Ethics Committee of the University of Wuerzburg. All patients providing colon cancer samples signed a consent form to allow for this research. The stage classification of the tumor tissue was determined according to the Union Internationale Contre le Cancer (UICC) system.

### Materials

PDGF-BB was purchased from Miltenyi Biotech (Bergisch Gladbach, Germany), VEGF-165 from R&D Systems (Minneapolis, MN, USA). InSolution™ Akt inhibitor IV, and PI3K inhibitor PKI-179 were obtained from Calbiochem, (San Diego, CA, USA). The Glucose Transporter Inhibitor IV, WZB117, was purchased from Merck Millipore (Darmstadt, Germany).

### Cancer cell lines and culture

The human colon cancer cell lines HT29, SW480, and HCT116 and the appropriate medium were obtained from ATCC (Manassas, VA, USA) and cells were cultured at 37°C in 5% CO_2_. HT29, and HCT116 cells were cultured in McCoy's 5A Medium, SW480 cells were cultured in RPMI medium, medium was supplemented with 10% (v/v) fetal bovine serum, and 1% (v/v) penicillin/streptomycin (both Life Technologies Carlsbad, CA, USA). We exemplarily used the HT29 colorectal cell line for most of the experiments. For representative substantiation, additionally, the SW480 and HCT116 cell line were used.

### Antibodies

For Western Blot immunostaining procedure, the membranes were incubated with Akt, phospho-Akt (Ser473) (D9E), phospho-S6 (Ser235/236) (D57.2.E), phospho-4E-BP1 (Thr37/46) (236B4), phospho-mTOR (Ser248) (D9C2), β-Actin (13E5), Cofilin (D3F9) (HRP conjugate), GAPDH (D16H11) (HRP conjugate), α-Tubulin (11H10) (HRP conjugate), phospho-Rb (Ser807/811), phospho-Rb (Ser795), phospho-p44/42 MAPK (Erk1/2) (Thr202/Tyr204) (D13.14.4E), phospho-MEK1/2 (Ser217/221) (all purchased from Cell Signaling, Beverly, MA, USA), anti-c-Myc (phospho S62), anti–Glucose Transporter GLUT1, and anti-MCT4 (both from abcam, Cambridge, UK). The blots were incubated with the HRP-conjugated secondary antibodies goat anti-rabbit IgG or goat anti-mouse IgG (both from Santa Cruz Biotechnology, Dallas, TX, USA).

### Methods

#### Quantitative real time polymerase chain reaction (real time PCR)

Gene expression of PDGF, VEGF, HIF1α, GLUT1, MCT4, LDHA, and KI67 was analyzed in colon cancer specimens by real time PCR. VEGF served as a control for the PDGF gene profiling. Complementary DNA (cDNA) was performed using the ImProm-II reverse transcriptase system (Promega, WI, USA) and an Eppendorf Mastercycler (Eppendorf, Hamburg, Germany). The TaqMan gene expression assays were purchased from Life Technologies. All samples were assayed in duplicate and normalized during data analysis. The housekeeping genes β-Actin, 18S rRNA, and RPLP0 (ribosomal protein lateral stalk subunit P0) were used for relative quantification, the relative quantification value is expressed as 2^−ΔΔCq^. Results were normalized to colon normal tissue purchased from Biochain (Hayward, CA, USA). PCR reactions were conducted with a BioRad CFX96 Touch real-time PCR detection system.

### Protein extraction and western blot analysis

Total protein extracts were assembled using RIPA lysis buffer Equal protein amounts (30-50 μg) were electrophoresed using NuPage Novex precast gels (Invitrogen/Life Technologies) and transferred with the iBlot dry blotting system (Invitrogen/Life Technologies). Bands were detected by ECL solution (Thermo Scientific, Waltham, MA, USA).

### MTS proliferation assay

The proliferation rate of cells was checked with the colorimetric CellTiter 96 Aqueous One Solution assay (Promega, WI, USA). 2500 cells/96well were seeded in each well. Cells were stimulated with VEGF, PDGF or VEGF and PDGF (100 ng/ml respectively) for 24 hours, 48 hours, and 72 hours under starving conditions.

For investigation of effect of the Akt inhibitor, cells were treated with VEGF, PDGF, or VEGF and PDGF and/or Akt inhibition (10 μM) for 30 minutes, 60 minutes and 24 hours. CellTiter 96^®^ AQueous One Solution Cell Proliferation Assay was added to each well and measured according to the manufacturer's instructions.

### Live/dead cell staining assay

The proportions of live and dead cells after treatment with PDGF (100 ng/ml), VEGF (100 ng/ml) or VEGF + PDGF and simultaneous incubation of Akt inhibitor (10 μM) or PI3K inhibitor (80 nM) for 2 hours were investigated with a PromoKine live/dead cell staining kit II (PromoCell, Heidelberg, Germany). The assay was performed according to the manufacturer's instructions, immunofluorescence was detected with an Olympus BX51 microscope and the CellSens Dimension software.

### ApopTag® Plus apoptosis assay

For apoptosis investigation, an ApopTag^®^ Plus Peroxidase *In Situ* Apoptosis detection assay (Merck Millipore) was performed. After incubation of HT29 cells with PDGF (100 ng/ml), VEGF (100 ng/ml) or VEGF + PDGF and simultaneous incubation of Akt inhibitor (10 μM) or PI3K inhibitor (80 nM) for 2 hours, cells were fixed with formaldehyde overnight. Staining procedure was performed according to the manufacturer's instructions. For immunofluorescence staining, a DyLight^®^594 Anti-Digoxigenin/Digoxin antibody (Vector, Burlingame, Ca, USA) and DAPI Fluoromount G mounting medium (Southern Biotech, Birmingham, Al, USA) was used. An Olympus BX51 microscope and the CellSens Dimension software was used for visualization, magnification x10.

### AnnexinV apoptosis assay

Cells treated with Akt inhibitor (10 μM) were incubated simultaneously with PDGF (100 ng/ml), VEGF (100 ng/ml) or PDGF + VEGF and detached with accutase solution (Sigma-Aldrich) 30 minutes, 60 minutes, 120 minutes, and 240 minutes after treatment. Cells were treated with AnnexinV-FITC Apoptosis Detection Kit (abcam) according to the manufacturer's instructions and analyzed on a flow cytometer (Beckman Coulter, Krefeld, Germany) with a software package (Coulter, Epics XL-MCL, System II).

### MILLIPLEX assay for human oxidative phosphorylation

Tumor protein extraction and procedure was performed according to the manufacturer's instructions (MILLIPLEX^®^ human oxidative phosphorylation (OXPHOS) magnetic bead panel; purchased from Merck Millipore). The protein concentration was 10 μg, sample preparations and data collection were performed according to the manufacturer's instructions. Values were given as median fluorescence intensity (MFI); n=2.

### DAB- and immunofluorescence staining

For DAB- and immunofluorescence staining, HT29 colon cancer cells were seeded on cover slips, fixed in formalin, and treated with the Dako EnVision™+ Dual Link System-HRP (DAB+) (Dako, Glostrup, Denmark) according to the manufacturer's instructions. For DAB and immunofluorescence staining PDGF monoclonal antibody was purchased from Santa Cruz Biotechnology, and VEGF monoclonal antibody was purchased from abcam. Secondary antibody was incubated for 60 minutes (DAB staining: rabbit/mouse polymer, part of Dako EnVision™+ Dual Link System-HRP (DAB+); immunofluorescence staining: goat anti mouse IgH H&L, Cy3, abcam; DAPI Fluoromount G mounting medium). An Olympus BX51 microscope and the CellSens Dimension software were used for visualization.

For immunofluorescence double staining, the colon cancer tissue slides were fixed in acetone and the antibodies bound during the first staining step were covered and fixed with Dako Doublestaining system, K1395 (Dako, Glostrup, Denmark), according to the manufacturer's instructions. The immunofluorescence double staining (coexpression) was detected with VEGF and PDGF, panCytokeratin monoclonal antibodies (both from Santa Cruz), and Vimentin (from abcam). Rabbit anti-mouse Cy3, and goat anti-rabbit Alexa488 secondary antibodies were purchased from dianova, Hamburg, Germany. An Olympus BX51 microscope and the CellSens Dimension software was used for visualization.

### Glucose transporter 1 inhibition

Cells were incubated for 24 hours with PDGF (100 ng/ml) and washed with PBS twice. After detaching the cells 1 × 10^6^ cells were treated with glucose and WZB117 and/or PDGF for 15 minutes. Glucose and Lactate Assay (both from abcam) were performed according to the manufacturer's instructions.

### Oxygen measurement

Oxygen consumption of HT29 cancer cells was measured with the Oxytherm system (Hansatech Instruments). The Oxytherm system was used in accordance with the manufacturer's instructions. After the calibration was conducted, 1 × 10^6^ treated or untreated cells in 1 ml media in each probe were measured with the Oxytherm device. Results after 0.5 minutes were evaluated with the Oxygraph plus software.

### Statistical analysis

Statistical analysis was performed using GraphPad Prism 5.0 (Graph Pad Software Inc., San Diego CA, USA). A Two-way ANOVA with Bonferroni post hoc test or a One-way ANOVA with post hoc tests according to sample distribution was used. Data were presented as mean ± standard deviation. p< 0.05 was considered to be statistically significant.

Quantification of Western Blot bands were analysed with Image Studio Lite. The relative densities of the bands were expressed as the percentage of the control.

## SUPPLEMENTARY MATERIALS FIGURES


