# Supplementary material for: Exclusive inhibition of PI3K/Akt/mTOR signaling is not sufficient to prevent PDGF-mediated effects on glycolysis and proliferation in colorectal cancer
Source: Oncotarget. 2016 Sep 8;7(42):68749–67. doi: 10.18632/oncotarget.11899 (PMC5356587; doi:10.18632/oncotarget.11899)
Supplement: Supplementary file 1 [file oncotarget-07-68749-s001.pdf]

Exclusive inhibition of PI3K/Akt/mTOR signaling is not sufficient to prevent PDGF-mediated effects on glycolysis and proliferation in colorectal cancer

SUPPLEMENTARY FIGURES

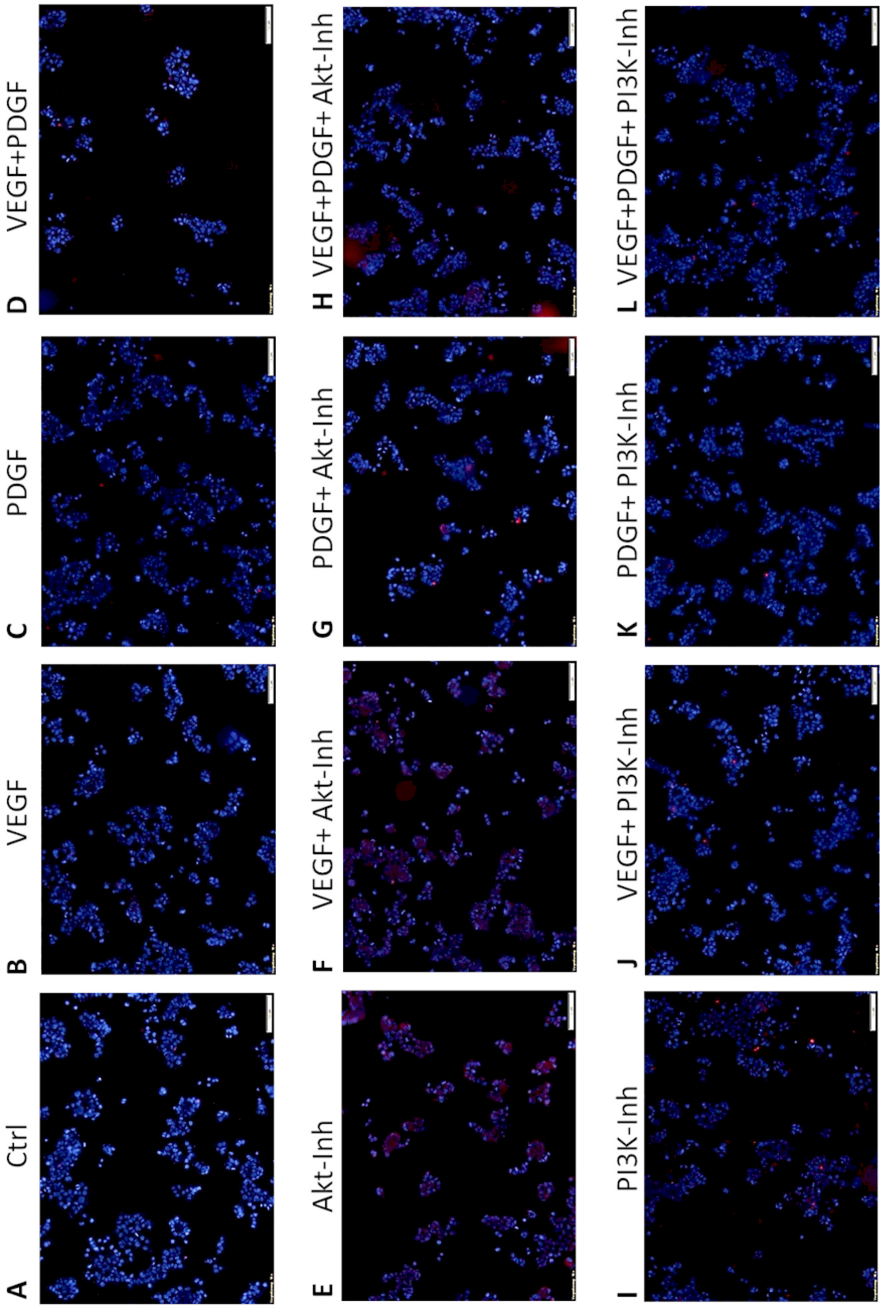

Supplementary Figure S1: Effect of PDGF stimulation and/or Akt and PI3K inhibition on apoptosis – ApopTag® Plus apoptosis assay A-L. Single treatment with PDGF (C), VEGF (B) or PDGF + VEGF (D) showed decreased amounts of apoptotic cells (red) compared to Akt inhibitor treatment (E). PDGF and VEGF (F-H) decreased apoptosis in presence of the Akt inhibitor (E). PI3K inhibitor marginally induced apoptosis, therefore PDGF and VEGF did not increase cell survival (I-L). Nuclear counterstaining with DAPI blue; magnification x10.

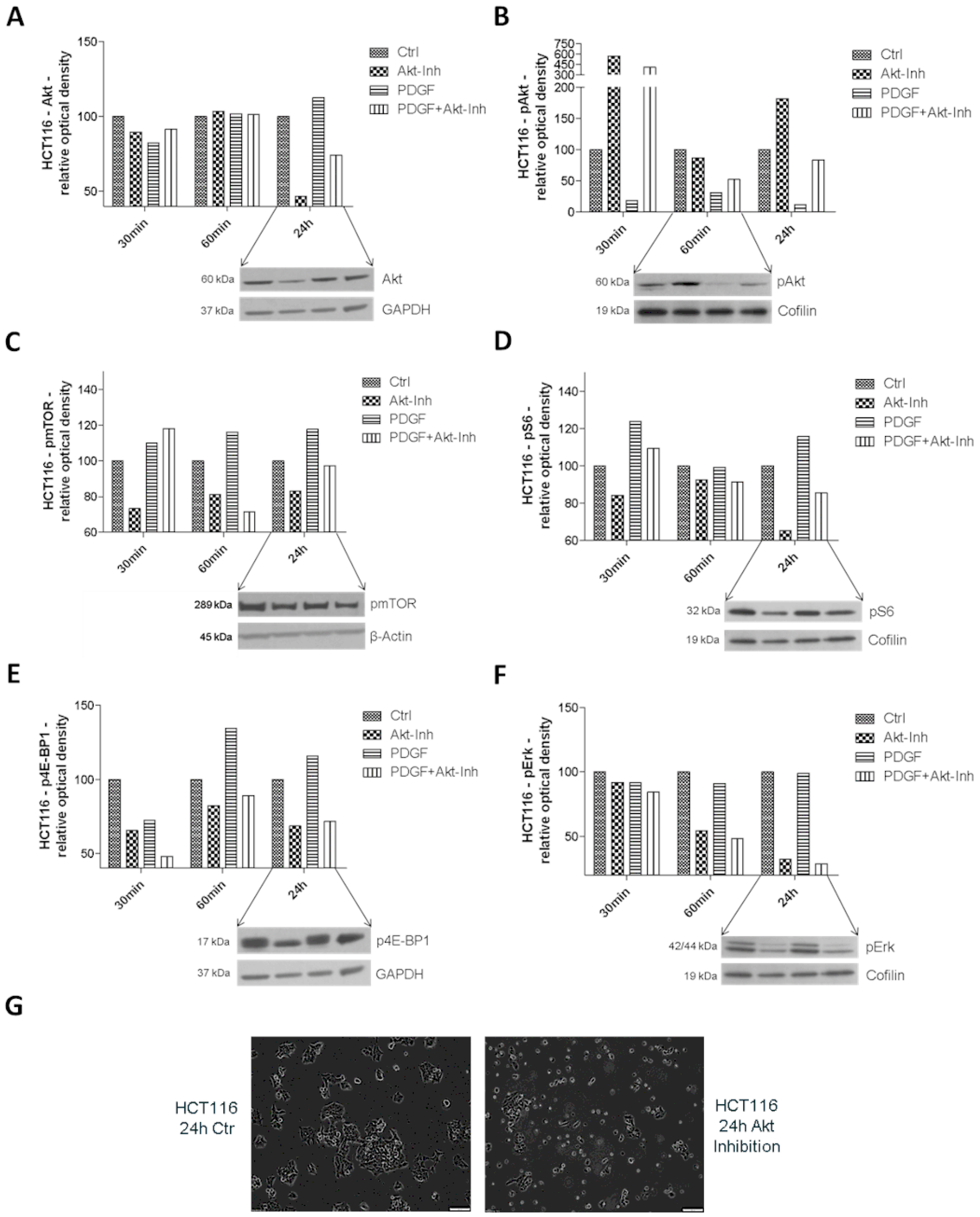

Supplementary Figure S2: Western Blot analysis showed the effects of PDGF stimulation and/or Akt inhibition on the PI3K/Akt/mTOR and MAPK pathway in HCT116 cells. Lower panels show the representative western blots. Upper panels show quantification of three independent western blot experiments of Akt **A.**, pAkt **B.**, pmTOR **C.**, pS6 **D.**, p4E-BP1 **E.**, and pErk **F.**, normalized to Actin, GAPDH or Cofilin loading control. Cells were treated with Akt Inhibitor IV (10 µM) or PDGF (100 ng/ml) and with both Akt inhibitor, and PDGF, n=1. **G.** show the antiproliferative effect of the Akt inhibition compared with untreated control cells.

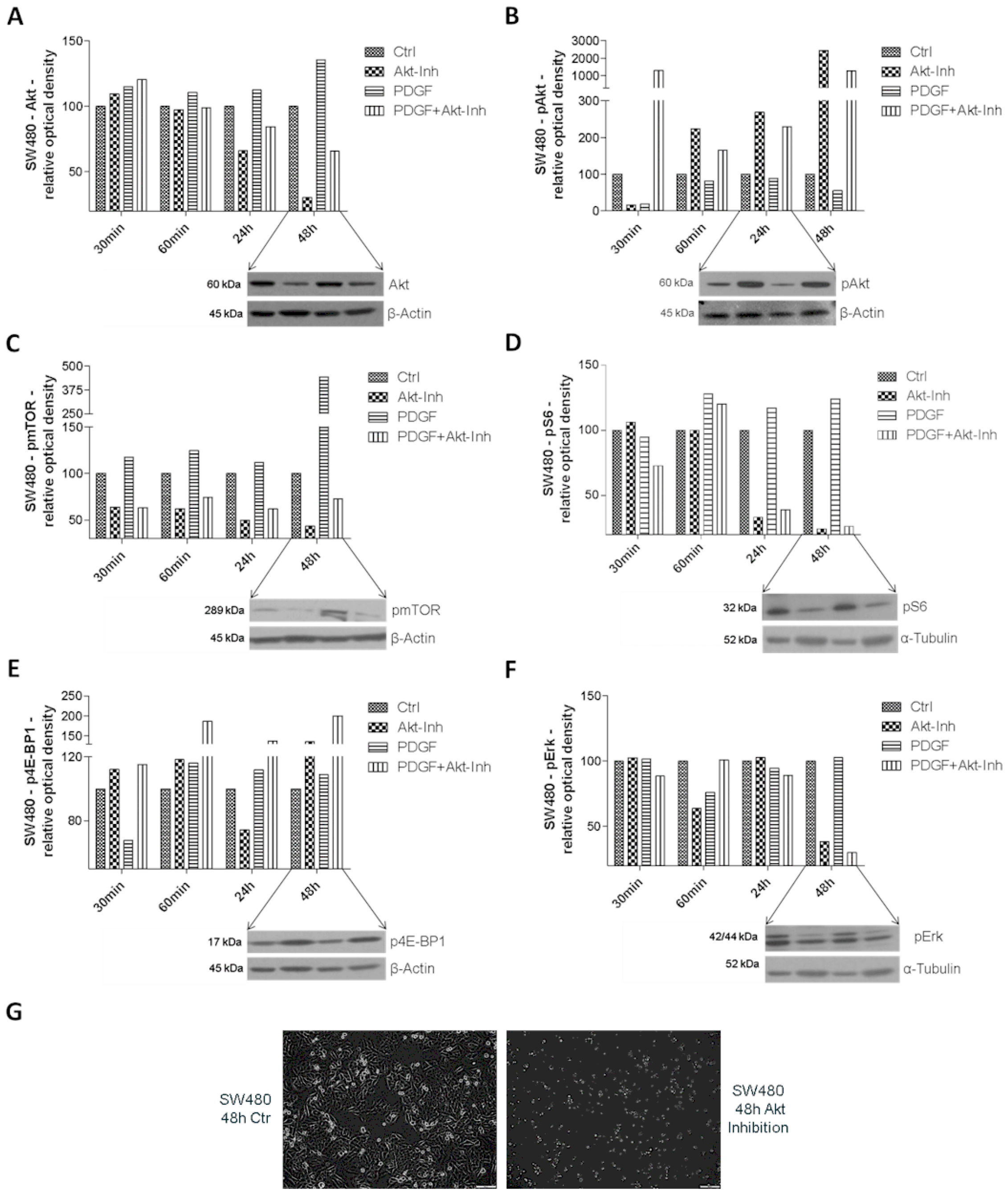

Supplementary Figure S3: Western Blot analysis showed the effects of PDGF stimulation and/or Akt inhibition on the PI3K/Akt/mTOR and MAPK pathway in SW480 cells. Lower panels show representative western blots. Upper panels show quantification of three independent western blot experiments of Akt **A.**, pAkt **B.**, pmTOR **C.**, pS6 **D.**, p4E-BP1 **E.**, and pErk **F.**, normalized to Actin or α-Tubulin loading control. Cells were treated with Akt Inhibitor IV (10 µM) or PDGF (100 ng/ml) and with both Akt inhibitor and PDGF, n=1. **G.** show the antiproliferative effect of Akt inhibition compared with untreated control cells.

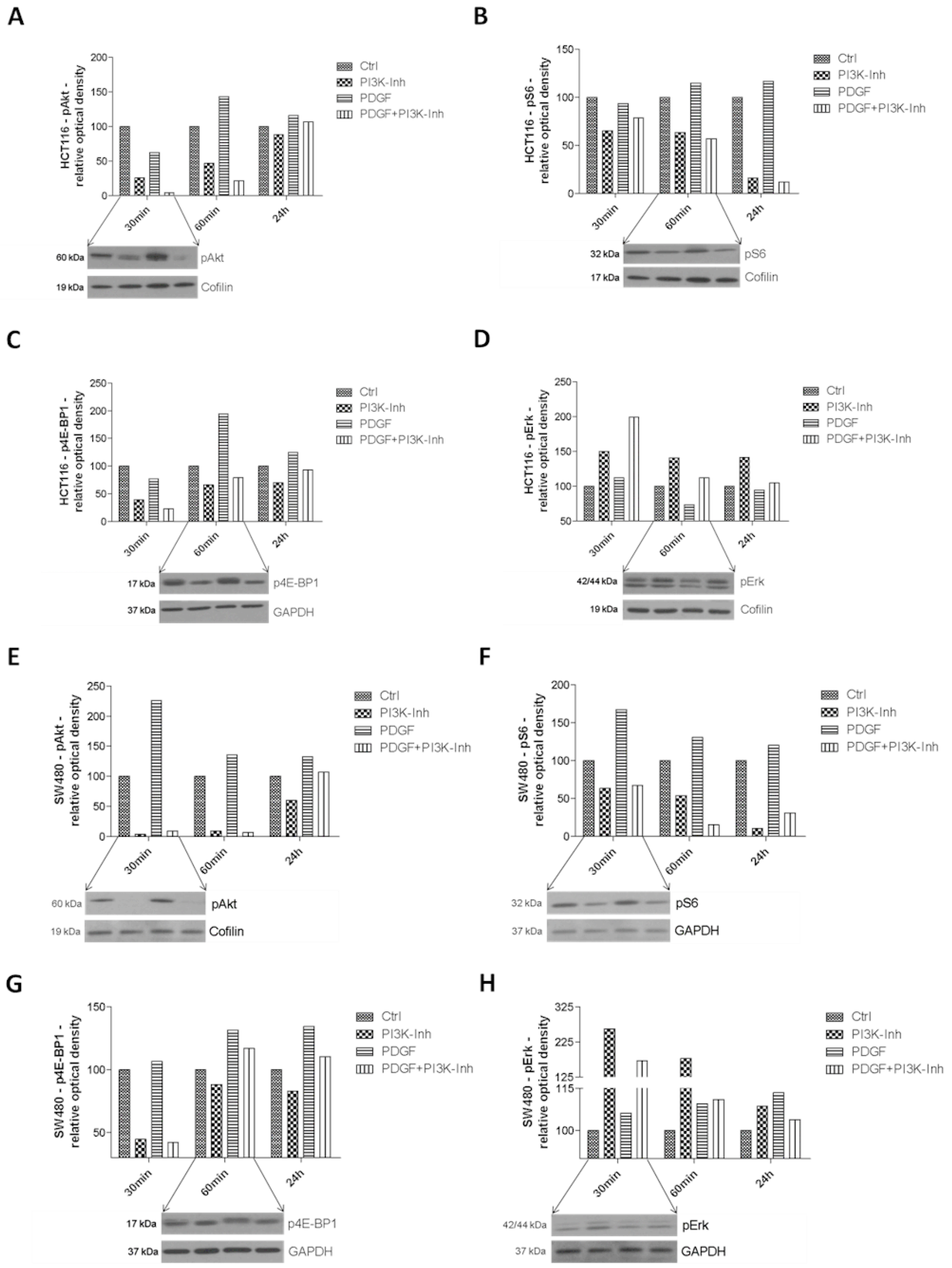

Supplementary Figure S4: Western Blot analysis representing the effects of PDGF stimulation and/or PI3K inhibition on the PI3K/Akt/mTOR and MAPK pathway in HCT116 and SW480 cells. Lower panels show representative western blots and upper panels show quantification of three independent western blot experiments of pAkt **A.**, pS6 **B.**, p4E-BP1 **C.**, and pErk **D.** in HCT116 cells and pAkt **E.**, pS6 **F.**, p4E-BP1 **G.**, and pErk **H.** in SW480 cells, normalized to GAPDH or Cofilin loading control. Cells were treated with PI3K Inhibitor (80nM) or PDGF (100 ng/ml) and with both PI3K inhibitor and PDGF, n=1.

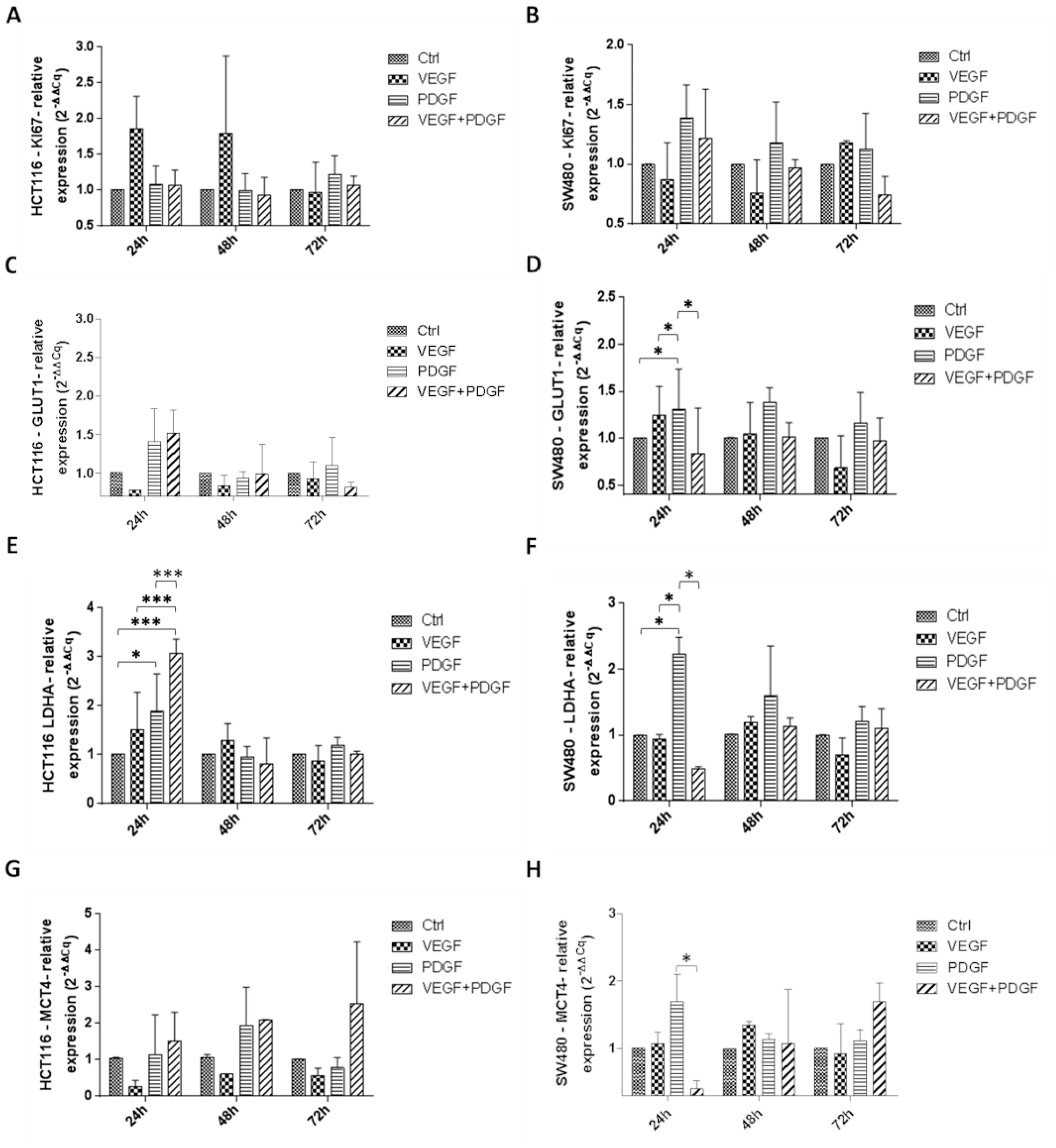

Supplementary Figure S5: Influence of PDGF on proliferation marker KI67 and glycolysis in HCT116 and SW480 cells. The proliferation marker KI67 was increased on gene level during PDGF, and VEGF stimulation in HCT116 **A.** and SW480 **B.** cells. Gene expression of the glycolysis markers GLUT1, LDHA, and MCT4 was increased in HCT116 **C-E.**, and SW480 **F-H.** Glycolysis markers were activated mainly during PDGF stimulation, but also during VEGF stimulation. Results were presented as ±SD. *p<0.05, ***p<0.001, n=3. Cells were treated with PDGF or VEGF, or both PDGF and VEGF (100 ng/ml respectively) for 24 hours, 48 hours, and 72 hours; n=3.
